# Effective Ethyl Carbamate Prevention in Red Wines by Treatment with Immobilized Acid Urease

**DOI:** 10.3390/foods13162476

**Published:** 2024-08-06

**Authors:** Elisa Tavilli, Marco Esti, Marcello Fidaleo

**Affiliations:** 1 Department for Innovation in Biological, Agro-Food and Forest Systems, University of Tuscia, 01100 Viterbo, Italy; 2 Department of Agriculture and Forest Sciences, University of Tuscia, 01100 Viterbo, Italy

**Keywords:** immobilized enzyme, enzyme deactivation, acid urease, stirred tank reactor, red wine

## Abstract

Climate change poses several challenges in the wine industry, including increasing risks related to chemical food contaminants such as biogenic amines and ethyl carbamate (EC). In this work, we focused on urea removal in red wines by immobilized acid urease aiming at limiting EC formation during wine storage. By considering separable kinetics of catalyst deactivation and urea hydrolysis, it was possible to model the time course of urea removal in repeated uses in stirred batch reactors. Treatments based on immobilized urease of red wine enriched with 30 mg/L of urea allowed the reduction in the contaminant concentration to <5 mg/L. After 28.5 h of treatment, the observed urea level was reduced to about 0.5 mg/L, corresponding to a decrease in the potential ethyl carbamate (PEC) from 1662 μg/L to 93 μg/L, below the level of the non-enriched wine (187 μg/L). As a comparison, when treating the same wine with the free enzyme at maximum doses allowed by the EU law, urea and PEC levels decreased to only 12 mg/L and 415 μg/L respectively, after 600 h of treatment. These results show that, for red wines, urease immobilization is an effective strategy for urea removal and, thus, effective reduction in ethyl carbamate as a process contaminant. This study provides the scientific background for the future scaling-up of the process at an industrial level.

## 1. Introduction

From a microbiological point of view, wines can be generally regarded as low-risk food products due to a relatively high ethanol content (9–14% *v*/*v*), along with low redox potential and acidity, and presence of polyphenols and sulfites [1], with the latter being either naturally occurring or added during winemaking. On the other hand, the presence of chemical hazards is much related to environmental conditions in vineyards (i.e., contamination by heavy metals, plant protection product residues, and mycotoxins from fungal growth), compounds forming in wines from microbial metabolisms (non-pathogens releasing toxic compounds), and residues deriving from technological aids and additives [1]. Storage conditions may exacerbate food contamination at concerning levels. Among food contaminants in wines, it is possible to include ethyl carbamate (EC). Classified as “probably carcinogenic to humans” [2], EC naturally occurs in wines through a spontaneous reaction between urea and ethanol during fermentation and under storage conditions [1]; high temperatures accelerate this reaction [1,3,4]. Concerning urea formation in wines, generally arginine is present in grapes in notable quantities; during fermentation, yeasts take arginine up as a nutrient and metabolize it, producing urea as an intermediate metabolite. Yeast cells can retain urea; however, when they can no longer accumulate it due to elevated concentrations, they release it in the fermentation medium. This is considered the main reaction leading to urea accumulation and, consequently, EC formation, although other pathways are recognized to contribute to a lesser extent (i.e., involving carbamoyl phosphate and citrulline) [1,4]. Therefore, relatively high ethanol and urea contents and wine-making conditions including warm temperatures and prolonged storage are of concern. A recent work consisted of a three-year study investigating the evolution of key ageing molecules, including EC, in Madeira wines produced as per the two typical methods (*estufagem* and *canteiro*) [5]. The authors observed differences in EC formation according to the grape variety used (e.g., Tinta Negra showed a higher predisposition to EC accumulation) and a strong correlation with ageing time. Moreover, a positive correlation between EC and all furans under study was observed.

Currently, climate change is a major challenge for the wine industry: its impacts on grapevine concern plant development and fruit composition. Temperature increases are affecting harvest time by bringing it forward in several wine-producing countries, with some further threats for certain wine styles [1]. The effects of higher temperatures include berry total acidity reduction, increased sugar content (thus subsequent increased wine ethanol content), and decoupled phenolic and technological maturity [6], with accumulation of sugars occurring before achieving phenolic maturation. Depending on the variety, edaphoclimatic conditions, and vineyard operations [7], since nitrogen (N) compounds, as well as arginine, accumulate during ripening, they can be expected to be higher in overripened grapes [1], also depending on the phenological stage at which grapes experience high temperatures [8]. Furthermore, when climate change forces producers to harvest grapes earlier, N-based fertilizers may be used to enhance volatile compound development in grapes, resulting in increased amino acid content, particularly arginine [1,9]. Thus, with higher alcohol content and/or arginine, the likelihood of facilitated EC formation may be awaited. 

Due to the abovementioned health concerns, the wine industry has a concrete interest to reduce EC formation. Several countries in the world have defined maximum allowable limits for EC in wines and alcoholic beverages (e.g., 30 μg/kg for table wines in Canada and Czech Republic, 100 μg/kg for fortified wines in Canada, and 60 μg/L for dessert wines in the US) [10]. Since urea represents the main precursor for EC formation reaction in wines, the key actions to prevent EC formation focus on reducing the presence of this precursor. Preventative strategies encompass best production practices both in vineyards and during winemaking: if properly managed, they would help minimize EC accumulation in finished products. Concerning vineyard management, increasing levels of N fertilization will proportionally augment N compound content in must and, consequently, in wine. For this reason, overall knowledge of soil status, cultivar and rootstock variety, cover crop impacts on N nutrition, and vine and grape nutritional status is recommended in order to reduce urea formation [4]. In winemaking, urea formation prevention can occur at different levels: (1) N compound composition and content in musts: yeasts require N to grow and ferment. Prior knowledge of N compounds in the must will be relevant to balance possible nutrient additions to avoid stuck/sluggish fermentation and to predict possible urea formation; (2) yeast strains: the ability to metabolize urea varies from strain to strain. For this reason, if arginine content in the must is too high, it is recommended to use low-urea-producing yeasts; (3) lactic acid bacteria (LAB): some strains can synthetize and excrete citrulline, another minor EC precursor, in small quantities. In addition to a balanced N status of the must, LAB strains for malolactic fermentation (MLF) should be carefully selected according to their ability to metabolize citrulline, and must citrulline concentration should be monitored throughout MLF; (4) use of urease to reduce urea residues when high [4]. Recently, scientific research has been working on metabolic engineering strategies to provide yeasts with low-urea-forming capabilities either by repression of urea production or enhanced urea metabolism [11]. Many successful attempts of improvement of wine yeasts feature genetic engineering tools [12]. An interesting work was carried out to obtain a genetically engineered yeast strain: along with no significant differences in fermentation performances if compared with the parental strains, the recombinant strains led to the reduction in both urea and EC in non-grape wine samples (−77.89 and −73.78%, respectively), whereas EC formation occurred at much lower rates during storage [13]. However, it is worth noting that there are still legal and cultural constraints to the use of genetically modified strains in the food industry despite their potential as technological tools [12,13,14]. 

The use of acid urease from *Lactobacillus fermentum* has been recognized as an effective strategy to reduce urea content, and it is currently permitted as an oenological practice [10]. Acid urease can be applied either as a free or immobilized biocatalyst. As previously pointed out [10], several constraints limit the application of the free biocatalyst (unfeasible recovery from treated bulk wine and reutilization, presence of naturally occurring compounds inhibiting its activity, etc.). In sake production, immobilized acid urease has been used in bioreactors since 1988 [10]. Concerning the wine industry, research on the use of immobilized acid urease from *L. fermentum* is recent. The immobilization protocol of this biocatalyst onto two acrylic resin supports was first developed in 2012 [15]. The procedure was effective to obtain high levels of enzyme immobilized per dry weight of resin, with a subsequent high activity and appreciable storage duration. The same authors assessed external mass transfer limitations on the biocatalyst activity [16,17], and a preliminary empirical rule was suggested for stirred or unstirred bioreactors design [17]. The two most recent scientific works on urea removal in real wines with immobilized acid urease focused, respectively, on using the biocatalyst in polyphenol-rich wines in a lab-scale packed bed reactor [18] and on the building of a model of the same bioreactor [19]. 

In red wines, free acid urease activity is notably reduced due to multiple inhibitors (ethanol, fluoride ions, malate, and some organic acids commonly present in wines) [3,10,20,21,22,23] and deactivating agents (wine polyphenols) [18], and this effect can be limited using immobilized urease [18]. To provide further knowledge on the use of immobilized acid urease in red wines, in this study, we focused on the application of such a biocatalyst carrying out urea hydrolysis in repeated uses in stirred batch reactors in a red wine. 

## 2. Fluidized Catalytic Reactor Model

### 2.1. Urea Hydrolysis Stoichiometry

Ureases (EC 3.5.1.5) catalyze the hydrolysis of urea into ammonia and carbon dioxide according to the following reaction equation:

(1) $$A\overset{urease,\;\;\;water}{\to }2\;B+C$$

where A, B, and C stand for (NH_2_)_2_CO, NH_3_, and CO_2_, respectively. 

### 2.2. Reaction Kinetics for Immobilized Urease

The intrinsic local urea formation rate $\left(r_{A}^{\prime }\right)$ for immobilized urease, referred to the catalyst mass, can be expressed through the Michaelis–Menten rate expression [17]:

(2) $$r_{A}^{\prime }=-\frac{k_{cat}Y_{P/B}C_{pA}}{K_{M}+C_{pA}}$$

where k_cat_ is the specific reaction rate constant, Y_P/B_ is the enzyme loading, K_M_ is the Michaelis constant, and C_pA_ refers to the concentration of urea inside the catalyst pellet. For C_pA_ << K_M_, Equation (2) reduces to a pseudo-first-order kinetics:

(3) $$r_{A}^{\prime }=-k_{1}C_{pA}$$

with k_1_ = k_cat_Y_P/B_/K_M_.

The corresponding observed urea formation rate is

(4) $$r_{A,obs}^{\prime }=-k_{1}C_{A}\Omega$$

where C_A_ is the bulk liquid concentration of urea, and Ω is the overall effectiveness factor [17,18].

### 2.3. Separable Kinetics of Catalyst Deactivation and Urea Hydrolysis

Irreversible deactivation of immobilized urease when stored in contact with rosé or red wine has been described by a biphasic kinetics [18] leading to the following equation for the ratio of the enzyme activity to the initial enzyme activity (f):

(5) $$f=\left(1-a\right)e^{-k_{d}t^{\prime }}+a$$

where t’ is the contact time between the catalyst and the wine, k_d_ is a first-order deactivation constant, and a is the ratio between the residual activity of the catalyst and its initial activity. 

By assuming separable kinetics of catalyst deactivation and urea hydrolysis, Equations (4) and (5) can be combined to obtain

(6) $$r_{A,obs}^{\prime }=-k_{1}C_{A}\;\Omega \left[\left(1-a\right)e^{-k_{d}t^{\prime }}+a\right]$$

### 2.4. Reactor Model

The concentration time course of urea in the reactor bulk liquid phase can be described by the mass balance equation for urea under non-stationary conditions:

(7) $$\frac{dC_{A}}{{dt}^{\prime }}=R_{A}$$

where t’ is time; C_A_ is the urea concentration in the liquid bulk; and R_A_, the mass transfer rate of urea from the liquid to the pellet per unit liquid volume, can be expressed as follows:

(8) $$R_{A}=r_{A,obs}^{\prime }\frac{W}{V}$$

where W is the mass of the catalyst, and V is the volume of the liquid phase. By substituting Equation (6) into Equation (8) and the resulting equation into Equation (7), we obtain

(9) $$\frac{dC_{A}}{{dt}^{\prime }}=-k_{1}C_{A}\;\Omega \left[\left(1-a\right)e^{-k_{d}t^{\prime }}+a\right]\frac{W}{V}=-k_{obs}C_{A}\;\left[\left(1-a\right)e^{-k_{d}t^{\prime }}+a\right]$$

where k_obs_ is a combined parameter, independent of urea concentration and time, equal to k_1_ Ω *W*/*V*. A multiphysics approach has been used to study the urease immobilized system employed in this work, and it demonstrated that it does not experience diffusion limitations when used in a white wine in a packed bed reactor [19]. This condition can be applied also for the stirred reactor under study, considering that diffusion limitations in red wines should be even less important compared with white wines, thanks to the lower activity of urease in red wines.

The time that appears in Equation (9) is the age of the catalyst. It can be expressed as t + t_0_, where t is the hydrolysis time of the current use, and t_0_ is the age of the catalyst from previous uses. Equation (9) can be integrated by variable separation with the initial condition C_A_(t = 0) = C_A0_ leading to

(10) $$ln\frac{C_{A}}{C_{A0}}={-k}_{obs}\left[\frac{\left(a-1\right)}{k_{d}}\left(e^{-k_{d}(t+t_{0})}-e^{-k_{d}t_{0}}\right)+at\right]$$

## 3. Materials and Methods

### 3.1. Raw Materials

A soluble powder containing acid urease from *Lactobacillus fermentum* was used (Nagapsin, lot n. 3021326; Nagase Europa GmbH, Duesseldorf, Germany). The activity of the powder, tested under standard conditions [19], was 379 ± 5 IU/g. Its protein content, estimated according to Bradford protein assay and expressed as bovine serum albumin equivalent (BSAE), was 4.8 ± 0.3% (*w*/*w*) [19].

A microporous, epoxy (oxirane)-activated acrylic support was used for immobilization (Eupergit^®^ C 250 L, lot no. B060519592; Röhm GmbH, Darmstadt, Germany) following the procedure previously described [15]. 

A red wine made with 60% Sangiovese and 40% Cabernet Sauvignon grapes and produced by Cantina Gentili (Cetona, SI, Italy) was used. Its main chemico-physical characteristics were reported in another work [3] and are available in Table 1.

The wine was collected from the maturation tank of the winery and stored in a 52 L bulbous narrow-necked bottle at 15 °C during the experimentation. All reagents used were of analytical grade.

### 3.2. Ammonium and Urea Determination

Urea and ammonium determination in wine was carried out using a K-URAMR kit (Megazyme International Ireland Ltd., Wicklow, Ireland). Wine samples of 250 µL were treated with 10 mg of polyvinylpyrrolidone (PVPP^®^), as previously described [18], before urea and ammonia determination. Ammonium ion concentration in buffer solutions was estimated using a Spectroquant Ammonium reagent kit (Merck KGaA, Darmstadt, Germany) according to procedures described in a previous work [18].

### 3.3. Potential Ethyl Carbamate Determination

The potential ethyl carbamate (PEC) is an index used to measure the susceptibility of wines to produce EC during storage. It is the concentration of EC measured in wine after an accelerated aging test. PEC was determined according to the procedure previously described [3]. Briefly, the wine was submitted to accelerated EC formation treatment by incubation at 71 °C for 48 h. The resulting ethyl carbamate concentration was measured by gas chromatography–mass spectrometry (GC-MS) as formerly described by the International Organisation of Vine and Wine, OIV [24] and referred to as potential ethyl carbamate [3]. 

### 3.4. Immobilized Enzyme Development and Characterization

The immobilized enzyme was produced and characterized as previously described [15]. Briefly, the immobilization buffer consisted of 1 M sodium phosphate buffer (pH 7.0), while the immobilization volume and the concentration of enzyme preparation were set to 100 cm^3^ and 50 g dm^−3^, respectively, with a mass of dry acrylic resin of 10 g dm^−3^. Processing temperature was kept constant at 25 °C. We incubated immobilization solution with the acrylic support in Falcon tubes, shaken for 48 h. After that, the obtained biocatalyst was collected and treated according to a specific washing procedure (with 0.05 M sodium phosphate buffer, neutral pH) and subsequently incubated in a 3 M glycine (pH 8.5) aqueous solution (24 h, 25 °C). We obtained beads of the immobilized biocatalyst, which were filtered and washed. The obtained beads exhibited a protein loading of 109.7 ± 2.4 mg of BSAE per g of dry beads and specific activity of 888 ± 20 IU per g of dry beads. The beads were stored in a storage buffer at 4 °C.

### 3.5. Urea Hydrolysis in the Stirred Bioreactor 

Urea hydrolysis experiments with the red wine were carried out in 50 mL adjustable hanging bar spinner flasks (Bellco Glass, Inc., Vineland, NJ, USA) to avoid any mechanical damage of the bead. A reaction time in the range 14.5–28.5 h was used. For this duration of the treatment, a temperature of 20 °C was chosen as a good balance between urease reactivity and wine preservation. Stirring speed was set at 320 rpm to ensure good mixing and reduce any external diffusion limitations. A water bath placed over a multistirrer (model Multistirrer 15, Velp Scientifica, Milan, Italy) was used to control the spinner flask temperature. Reactors were run under the following conditions: wine volume, 50 mL; bead mass, 270 mg of wet bead; and urea concentration in wine adjusted to 30 mg/L. The wine sample was poured into the spinner flask and allowed to reach steady levels for reaction temperature (20 °C) before being inoculated with the beads. Several samples (250 μL) were withdrawn from the wine as a function of time and assayed for the residual urea and ammonia contents. The immobilized biocatalyst was stored in contact with red wine at 4 °C for less than 12 h. After that period, it was possible to recover the beads through filtration and washing. The catalyst was, therefore, used for the second cycle of urea hydrolysis with fresh red wine enriched with urea. Sampling and incubation were repeated as described above. 

### 3.6. Experimental Design

Two sets of urea hydrolysis experiments were run. The first set aimed at modelling the urea hydrolysis rate in repeated uses of the immobilized biocatalyst. It consisted of two subsequent experiments in spinner flask reactors: the first one was carried out with fresh beads and was run for 24 h, while the second one was carried out using the catalyst recovered from the first experiment and restored as reported above. The second set was designed to assess the effectiveness of such immobilized urease treatments to prevent EC formation through urea hydrolysis by running accelerated EC formation tests and measurements and to validate the developed model. To this end, two hydrolysis experiments were run to treat the red wine enriched with urea (concentration of additional urea: 30 mg/L) for 14.5 or 28.5 h. Such treatment times were obtained by using the developed mathematical model by imposing desired final urea levels.

### 3.7. Statistical Analysis

Experiments were mostly nonreplicated and were analyzed by linear or nonlinear regression approaches using Microsoft^®^ Excel^®^ spreadsheet software (for Microsoft 365 MSO, version 2406, build 16.0.17726.20078) and its built-in solver. For replicated data, three replicates were considered, and the results were expressed as mean ± standard deviation.

## 4. Results and Discussion

**Figure 1:**
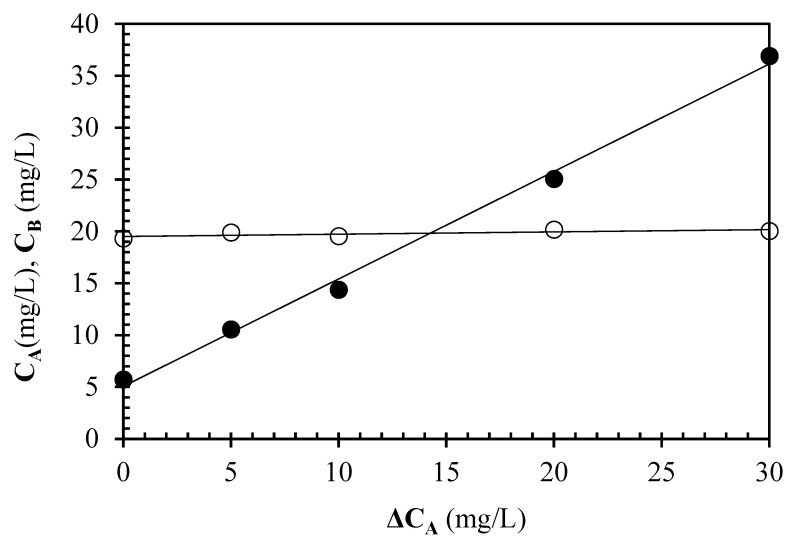
Effect of urea concentration additions (ΔC_A_) on measured urea (C_A_, ●) and ammonia (C_B_, ○) concentrations in the red wine.

The results of urea and ammonia determinations in the red wine undergoing urea hydrolysis are reported in Figure 1. Since samples were used undiluted, and subjected to PVPP treatment, the test accuracy was assessed by the method of standard additions. From the data plotted in Figure 1, it was possible to estimate the urea and ammonia content of the non-enriched wine as 5.1 ± 0.7 mg/L and 19.9 ± 0.3 mg/L, respectively. As an evident consequence of additional amounts of urea, the urea concentration (C_A_) in wine samples linearly increased, matching urea additions, while there was no effect on the ammonia concentration (C_B_), which remained constant at increasing levels of urea. Thus, the analytical quality of the data was confirmed.

**Figure 2:**
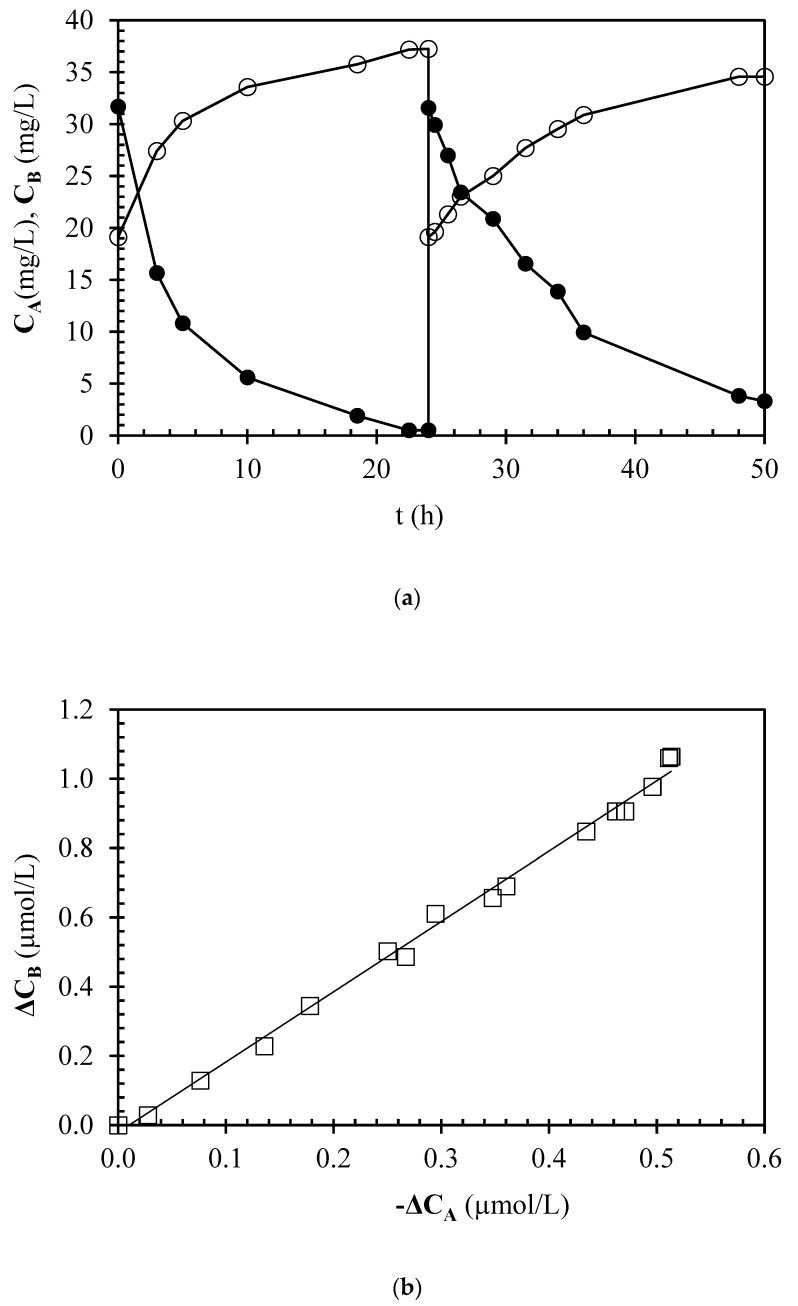
Repeated bioconversions in the stirred batch reactor: concentrations of urea (C_A_, ●) and ammonia (C_B_, ○) as a function of time (t) (**a**); increase in ammonia concentration (ΔC_B_) as a function of decrease in urea concentration (−ΔC_A_) (□) (**b**).

The data reported in Figure 2 show the results of urea hydrolysis catalyzed by the immobilized enzyme in two repeated cycles. From Figure 2a, it is evident that the hydrolysis rate is maximum at the beginning of the process, with the curves showing larger positive or negative slopes of the tangent straight lines at the initial time for ammonia or urea concentrations, respectively. Then, the rates decline as a function of time in a manner similar to a first-order kinetics. However, when the biocatalyst is reused, both the initial and final rates of reaction are lower compared with the first cycle. This reduction in activity can be attributed to catalyst deactivation. As can be seen in Figure 2b, the data for ammonia and urea concentration are consistent, satisfying the stoichiometry constraint, the slope of the ammonia concentration increment vs. the urea concentration decrement being equal to 1.98 ± 0.02, thus almost coincident with the ratio of the stoichiometry coefficient of ammonia to urea (equal to 2).

**Figure 3:**
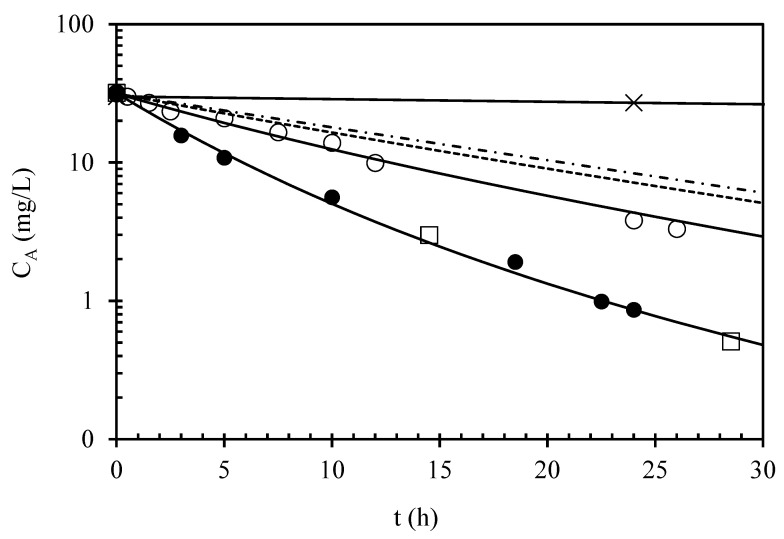
Effect of urea consumption and urease deactivation on the time course of urea concentration (C_A_). Symbols refer to the first use of the immobilized catalyst (●) or to the reuse after having been used for 24 h (○). The continuous line was obtained by fitting the data of the first and second cycles of use by using Equation (10). The square symbols (□) refer to the final urea content in the two hydrolysis batches used to determine PEC; however, their corresponding values were not used in the fitting procedure. The dashed and dash-dotted lines refer to simulations based on Equation (10) of urea hydrolysis at the hypothetical third and fourth 24 h cycles of reuse, respectively. The continuous, virtually horizontal line (✕) corresponds to urea hydrolysis occurring with the use of free urease according to the maximum dosage (375 IU/L, corresponding to 150 mg of enzyme preparation per liter of wine treated) allowed by the EU law [25] assessed in a previous work on the same red wine [3].

The urea concentration data are reported as a function of time in the semilogarithmic plot of Figure 3. It is possible to observe that there is no overlap between hydrolysis curves for the first and second cycles. This evidence reflects the catalyst deactivation effect mentioned above, which caused a reduction in the activity. Furthermore, the trend of the data does not appear linear, proving that they cannot be assessed by using a first-order kinetics model. By using the model described by Equation (10), which includes both catalyst deactivation and urea hydrolysis, together with the deactivation data determined by [18] (a = 0.24, k_d_ = 0.049 h^−1^), it was possible to estimate just one parameter, k_obs_, as equal to 0.22 h^−1^. The model appreciably fitted the data, as shown by the two continuous lines for the first and the second treatment cycles in Figure 3. The model and the estimated value of k_obs_ were used to calculate the hypothetical time course of urea for the third and fourth 24 h cycles of use. The corresponding lines appear linear on the semilogarithmic plot of Figure 3, thus confirming that when the catalyst is not deactivating, or is slowly deactivating, urea kinetics at low urea concentration (<30 mg/L) is of pseudo-first order. With reference to a previous study [3], where the authors studied urea hydrolysis by free urease in white, rosé, and red wines, this concomitant deactivation of urease is probably the reason why the first-order kinetics model fitted well the data obtained for white (R^2^ = 0.996, 0.990, 0.972) and rosé (R^2^ = 0.987) wines without any apparent deviation from the model, but was less suitable for the two red wines investigated (R^2^ = 0.975, 0.974), showing a clear departure from the linear trend in the semilogarithmic plot. 

The developed model was employed to design two urea hydrolysis treatments starting from a wine containing 31.7 mg/L of urea to obtain the desired final levels of urea of 2.63 and 0.55 mg/L. The treatment times predicted by the model were 14.5 and 28.5 h, respectively. The experimental values for the urea concentrations, reported in Figure 3, were 3.0 and 0.5 mg/L, thus in very close agreement with the model predictions. 

**Figure 4:**
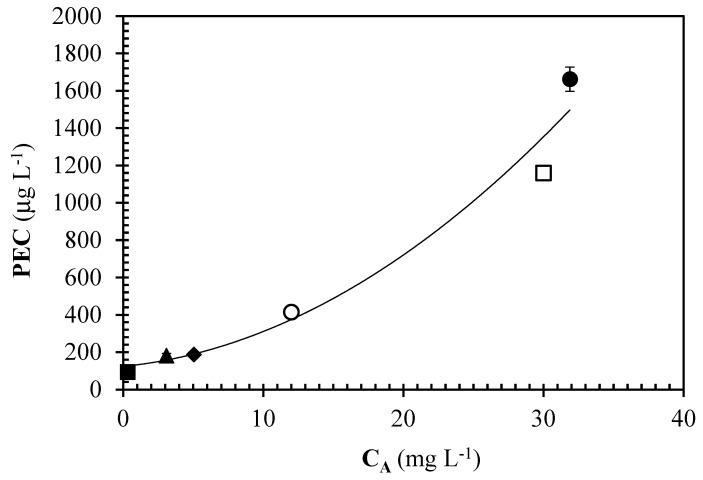
Effect of free [3] or immobilized urease treatments on potential ethyl carbamate (PEC) of red wine as a function of residual urea concentration (C_A_). The closed symbols refer to the red non-enriched wine (♦), enriched with urea at 30 mg/L (●) or enriched with urea and treated with immobilized urease for 14.5 (▲) or 28.5 h (■). The data obtained with the free enzyme refer to the red wine enriched with 30 mg/L of urea (□) and treated for 600 h at a urease dose of 375 IU/L (as set by EU law [25]) (○). Bars represent standard deviations of three replicates.

The EU law authorizing the use of acid urease from *L. fermentum* for oenological purposes sets, as field of application of this enzyme, the hydrolysis of urea when it is initially present at concentrations above 1 mg/L in wines that will undergo extended ageing [25]. In addition to the assessment of the efficacy of the immobilized catalyst in stirred batch reactor to remove urea, we assessed its efficacy to prevent EC formation as well. To this purpose, four samples of red wine, differently treated, were considered and subjected to accelerated EC formation: two samples were collected from urea-enriched (final urea concentration: 31.7 mg/L) red wine treated in a stirred batch reactor with an immobilized catalyst for 14.5 h or 28.5 h, respectively. The third sample of red wine corresponded to untreated red wine, enriched with 30 g/L of urea. Concerning the fourth sample, we considered data previously reported [3] for the same red wine, enriched with urea (additional urea at 30 mg/L) and treated with free acid urease at maximum allowed dosage. This allows making comparisons between the efficacy of urea hydrolysis carried out by immobilized acid urease in laboratory stirred batch conditions and laboratory use of the free catalyst to prevent EC formation in real red wine. Results are plotted in Figure 4 as a function of urea concentration for the samples we assayed including also the non-enriched wine. 

The PEC value of each wine was found to be proportional to the urea level, as expected. The two treatments studied were able to reduce the PEC of the enriched wine to levels that were lower than the one of the non-enriched wine. It is worth noting that the treatment with the maximum allowed dose of free urease in the red wine enriched with 30 mg/L of urea resulted in residual urea and PEC of 12 mg/L and 415 μg/L, respectively, after 600 h of hydrolysis [3]. Thus, it can be stated that immobilized urease can effectively remove urea in red wines and decrease the formation rate of EC. Interestingly, the approximating continuous quadratic polynomial reported in Figure 4 does not pass through the origin, indicating that even by totally removing urea, a small amount of PEC would still be determined. This may be because of other precursors of EC in red wines, even though urea remains the main contributor to EC formation in wine, as discussed above. 

Looking at the potential application of immobilized acid urease from *L. fermentum* from a wine industry’s perspective, it is worth noting that, compared with the hydrolysis occurring through free enzyme, the immobilized biocatalyst can effectively prevent EC formation in wines and other alcoholic beverages. The red wine investigated here was enriched with high levels of urea (30 mg/L): in real table wines, urea is generally present at concentrations ranging from 1 to 3 mg/L on average [26,27]. In the first cycle (Figure 2a), our fresh biocatalyst was able to reduce urea at levels below 1 mg/L (threshold set by the EU for the application of acid urease in red wines intended for long ageing periods), thus showing potential to prevent EC formation at levels far beyond those set by international laws. In some cases, maximum allowable levels of EC in wines are quite low (e.g., 30 µg/kg for table wines marketed in Canada) [10]. Moreover, from a processing point of view, using immobilized catalysts such as the one assessed here may help winemakers overcome some processing constraints occurring with the use of free acid urease: (1) the data reported here clearly show how processing times can be significantly decreased compared with the data reported in the literature for free acid urease in red wines [3]; (2) the catalyst can be recycled for subsequent treatments considering residual activity; (3) it is possible to work at higher enzyme doses of the catalyst, in compliance with relevant legislation, limiting the food safety risks mainly associated to allergies for the presence of enzyme residues since the enzyme would be covalently bound to the support, thus skipping specific filtration operations to remove free urease. 

## 5. Conclusions

This work demonstrated that the rate of urea hydrolysis by immobilized acid urease is determined by independent first-order hydrolysis kinetics and biphasic catalyst deactivation. A simple model considering the abovementioned phenomena and neglecting diffusion effects was successfully used to reconstruct hydrolysis data from repeated catalyst use. Opposite to the treatment of red wines with free urease, the use of the immobilized enzyme proved to be effective in reducing urea concentration and the corresponding ethyl carbamate formation. The ability of the catalyst to keep a residual activity even after 170 h of contact with the red wine makes immobilization of urease a possible strategy to treat red wines at a high enzymatic dose. Considering the emerging risks associated with the likelihood of EC formation in red, particularly prolongedly aged wines, because of the effects of climate change on wine grapes, this work provides further knowledge for the implementation of an effective EC prevention strategy at an industrial scale. 

## Figures and Tables

**Table 1:** Chemical and physical characteristics of the 60% Sangiovese and 40% Cabernet Sauvignon red wine used in this work [3].

Parameter	Unit	Value
Ethanol	% *v*/*v*	12.5 ± 0.1
pH	-	3.66 ± 0.03
Total acidity	g TAE/L	5.3 ± 0.1
Volatile acidity	g AAE/L	0.76 ± 0.06
Tartaric acid	g/L	1.8 ± 0.2
L (−) Malic acid	g/L	0.1 ± 0.3
L (+) Lactic acid	g/L	1.4 ± 0.3
Citric acid	g/L	0.47 ± 0.05
Total SO_2_	mg/L	93 ± 3
Free SO_2_	mg/L	18.0 ± 0.5
Total phenols	mg GAE/L	1663 ± 25
Total polyphenol index	-	36.9 ± 0.3

## Data Availability

The original contributions presented in the study are included in the article, further inquiries can be directed to the corresponding author.

## References

[1] Ubeda C., Hornedo-Ortega R., Cerezo A.B., Garcia-Parrilla M.C., Troncoso A.M. (2020). Chemical Hazards in Grapes and Wine, Climate Change and Challenges to Face. Food Chem..

[2] IARC (2010). IARC Monographs on the Evaluation of Carcinogenic Risks to Humans.

[3] Cerreti M., Fidaleo M., Benucci I., Liburdi K., Tamborra P., Moresi M. (2016). Assessing the Potential Content of Ethyl Carbamate in White, Red, and Rosé Wines as a Key Factor for Pursuing Urea Degradation by Purified Acid Urease. J. Food Sci..

[4] (2024). FDA Information on Ethyl Carbamate (Urethane) in Foods and Beverages. https://www.fda.gov/food/chemicals/ethyl-carbamate-preventative-action-manual.

[5] Pereira V., Leça J.M., Freitas A.I., Pereira A.C., Pontes M., Albuquerque F., Marques J.C. (2022). Unveiling the Evolution of Madeira Wine Key Metabolites: A Three-Year Follow-Up Study. Processes.

[6] Santos J.A., Fraga H., Malheiro A.C., Moutinho-Pereira J., Dinis L.-T., Correia C., Moriondo M., Leolini L., Dibari C., Costafreda-Aumedes S. (2020). A Review of the Potential Climate Change Impacts and Adaptation Options for European Viticulture. Appl. Sci..

[7] Gutiérrez-Gamboa G., Alañón-Sánchez N., Mateluna-Cuadra R., Verdugo-Vásquez N. (2020). An Overview about the Impacts of Agricultural Practices on Grape Nitrogen Composition: Current Research Approaches. Food Res. Int..

[8] Moukarzel R., Parker A.K., Schelezki O.J., Gregan S.M., Jordan B. (2023). Bunch Microclimate Influence Amino Acids and Phenolic Profiles of Pinot Noir Grape Berries. Front. Plant Sci..

[9] Gutiérrez-Gamboa G., Garde-Cerdán T., Carrasco-Quiroz M., Martínez-Gil A.M., Moreno-Simunovic Y. (2018). Improvement of Wine Volatile Composition through Foliar Nitrogen Applications to ‘Cabernet Sauvignon’ Grapevines in a Warm Climate. Chil. J. Agric. Res..

[10] Tavilli E., Fidaleo M. (2024). Ureases in the Beverage Industry. Ureases.

[11] Zhao X., Du G., Zou H., Fu J., Zhou J., Chen J. (2013). Progress in preventing the accumulation of ethyl carbamate in alcoholic beverages. Trends Food Sci. Technol..

[12] Shalamitskiy M.Y., Tanashchuk T.N., Cherviak S.N., Vasyagin E.A., Ravin N.V., Mardanov A.V. (2023). Ethyl Carbamate in Fermented Food Products: Sources of Appearance, Hazards and Methods for Reducing Its Content. Foods.

[13] Guo X.W., Li Y.Z., Guo J., Wang Q., Huang S.Y., Chen Y.F., Du L.P., Xiao D.G. (2016). Reduced production of ethyl carbamate for wine fermentation by deleting CAR1 in *Saccharomyces cerevisiae*. J. Ind. Microbiol. Biotechnol..

[14] Grossmann M., Kießling F., Singer J., Schoeman H., Schröder M.B., von Wallbrunn C. (2011). Genetically modified wine yeasts and risk assessment studies covering different steps within the wine making process. Ann. Microbiol..

[15] Bortone N., Fidaleo M., Moresi M. (2012). Immobilization/Stabilization of Acid Urease on Eupergit^®^ Supports. Biotechnol. Prog..

[16] Bortone N., Fidaleo M., Moresi M. (2013). Assessment of Diffusion Limitations on the Performance of Immobilised Acid Urease Derivatives. Chem. Eng. Trans..

[17] Bortone N., Fidaleo M., Moresi M. (2014). Internal and External Mass Transfer Limitations on the Activity of Immobilised Acid Urease Derivatives Differing in Enzyme Loading. Biochem. Eng. J..

[18] Fidaleo M., Tavilli E. (2021). Urea Removal in Rosé and Red Wines by Immobilised Acid Urease in a Packed Bed Reactor. Food Bioprod. Process..

[19] Mazzù R., Tavilli E., Fidaleo M. (2023). Experimental Study and Modeling of a Packed Bed Bioreactor for Urea Removal in Wines. Food Bioprod. Process..

[20] Trioli G., Ough C.S. (1989). Causes for Inhibition of an Acid Urease from *Lactobacillus fermentus*. Am. J. Enol. Vitic..

[21] Famuyiwa O.O., Ough C.S. (1991). Modification of Acid Urease Activity by Fluoride Ions and Malic Acid in Wines. Am. J. Enol. Vitic..

[22] Matsumoto K., Tanaka A., Tosa T., Kobayashi T. (1993). Removal of urea from alcoholic beverages by immobilized acid enzymes.. Industrial Application of Immobilized Biocatalysts.

[23] Kodama S. (1996). Optimal conditions for effective use of acid urease in wine. J. Food Sci..

[24] Gaetano G., Matta M. (1987). Determination of Ethyl Carbamate in Wines and Spirits by Gas Chromatography and Mass Spectrometry. Bull. l’OIV.

[25] European Commission (2019). Commission Delegated Regulation (EU) 2019/934 of 12 March 2019 Supplementing Regulation (EU) No 1308/2013 of the European Parliament and of the Council as Regards Wine-Growing Areas Where the Alcoholic Strength May Be Increased, Authorised Oenological Pra. http://data.europa.eu/eli/reg_del/2019/934/oj.

[26] Ribéreau-Gayon P., Glories Y., Maujean A., Dubourdieu D. (2006). Nitrogen Compounds. Handbook of Enology.

[27] Francis P.S. (2006). The Determination of Urea in Wine—A Review. Aust. J. Grape Wine Res..

